# Analysis of the early clinical outcomes of arthroscopic debridement in the treatment of shoulder tuberculosis

**DOI:** 10.1186/s13018-020-02086-7

**Published:** 2020-11-20

**Authors:** Yanwei He, Juncai Liu, Zhi Wang, Peng Zhou, Xiangtian Deng, Li Yang, Zan Chen, Zhong Li

**Affiliations:** 1 Department of Orthopaedics, The Affiliated Hospital of Southwest Medical University, Lu Zhou, Si Chuan Province People’s Republic of China; 2 School of Medicine, Nankai University, Tian Jin, People’s Republic of China; 3 Department of Hematology, The Affiliated Hospital of Southwest Medical University, Lu Zhou, Si Chuan Province People’s Republic of China

**Keywords:** Shoulder, Tuberculosis, Arthroscopy, Treatment

## Abstract

**Background:**

Due to atypical clinical symptoms, it is difficult to diagnose joint tuberculosis infection, which often results in misdiagnosis and missed diagnosis. It is easy to cause joint disability. And there are few reports of using arthroscopy to diagnose and treat shoulder tuberculosis. This case series aims to introduce the clinical outcomes of arthroscopic treatment of shoulder tuberculosis.

**Methods:**

Twenty-nine patients with shoulder tuberculosis from September 2013 to February 2019 were included (10 males, 19 females; age range from 22 to 69; the average age is 37.6 years). All patients underwent arthroscopic lesion debridement, with preoperative and postoperative regular use of isoniazid, rifampicin, pyrazinamide, and streptomycin quadruple anti-tuberculosis drugs. The erythrocyte sedimentation rate (ESR) and C-reactive protein (CRP) were recorded before and at the last follow-up. The shoulder function was evaluated according to the visual analogue scoring method (visual analogue scale, VAS) pain score and Constant score.

**Results:**

Twenty-nine patients were followed up from 12 months to 2 years, and the average follow-up time was 15.7 months. The pathological diagnosis of all patients after surgery was shoulder tuberculosis. No serious complications were found at the last follow-up, and the incision healed well. VAS pain score, Constant score, ESR, and CRP at the last follow-up were significantly improved compared with those before treatment (*P* < 0.05).

**Conclusion:**

On the basis of the standard use of anti-tuberculosis drugs before and after surgery, shoulder arthroscopy is used to treat early and mid-term shoulder tuberculosis, which can be diagnosed by direct observation under the arthroscope and postoperative pathological examination. It has the advantages of thorough lesion removal, minimal invasiveness, rapid recovery, and reliable clinical effect.

## Background

Tuberculosis (TB) is a chronic, granulomatous, and necrotizing disease caused by infection with *Mycobacterium tuberculosis* (MTB) [1]. Tuberculosis has always been one of the world’s public health problems. According to statistics from the World Health Organization, there were approximately 6.4 million new tuberculosis cases worldwide in 2018. There were 866,000 new cases in China in 2018 [2, 3]. Osteoarticular tuberculosis accounts for about 10% of extrapulmonary tuberculosis [3]. The shoulder joint is a rare part of joint tuberculosis [4]. With the increase of drug-resistant tuberculosis strains and patients with autoimmune diseases, the incidence of shoulder tuberculosis has also increased [3, 5–7]. Shoulder tuberculosis can cause bone and joint damage, seriously affecting the ability to move and the quality of life [8]. Because of its atypical symptoms, shoulder tuberculosis is difficult to obtain etiological evidence. It is difficult to distinguish from malignant tumors, chronic infections, and other diseases in imaging, which makes the diagnosis difficult, so it is often missed and misdiagnosed, and the disability rate is very high [9]. After years of popularization and development, shoulder arthroscopy technology has become one of the important ways to solve shoulder diseases with its advantages of small impact on anatomical structures, minimal invasiveness, and rapid recovery [10, 11]. Clinicians can make a preliminary diagnosis through microscopic observation and then obtain specimens for further confirmation. Arthroscopy can remove inflammatory factors and necrotic bone and improve postoperative joint function. This article retrospectively analyzed 29 cases of minimally invasive treatment of tuberculosis under shoulder arthroscopy performed in our unit. The clinical outcomes are satisfactory. The report is as follows.

## Methods

Inclusion criteria include (1) patients with regular anti-tuberculosis treatment for more than 3 weeks before surgery, (2) patients with joint tuberculosis diagnosed pathologically after surgery, (3) patients treated with arthroscopy, and (4) patients who take anti-tuberculosis drugs regularly after surgery.

Exclusion criteria include (1) patients who received conservative treatment and did not receive arthroscopy; (2) in addition to arthroscopy, patients who need other surgical procedures; (3) patients who fail to take anti-tuberculosis drugs on time and regularly as prescribed by a doctor; and (4) patients with infectious active tuberculosis.

From September 2013 to February 2019, a total of 29 patients met the inclusion criteria, 10 males and 19 females, age range from 22 to 69 years, with an average age of 37.6 years. All patients had single joint involvement of the shoulder joint. Eleven patients had a history of tuberculosis. The average course of the disease is 13 months (7–24 months). All patients undergo detailed clinical, laboratory, and imaging examinations before surgery, including blood routine examination, liver and kidney function, X-ray, CT, (SPE/CT) MRI, tuberculosis T-SPOT examination, and other routine examinations. All patients had elevated ESR and CRP before surgery. No sinus formation was seen in all patients. All patients should be prescribed anti-tuberculosis drugs before surgery and be treated for at least 3 weeks according to the principles of regularity, whole course, combination, and appropriate amount. The drugs included isoniazid, rifampicin, pyrazinamide, and ethambutol in a quadruple anti-tuberculosis treatment, with supportive treatment. When the symptoms of tuberculosis poisoning have basically disappeared, the systemic symptoms and nutritional status have basically improved, and other basic diseases had stabilized, the preoperative erythrocyte sedimentation rate (ESR) is adjusted to below 40 mm/h, and surgical treatment can be arranged.

### Surgical purpose and technique

All operations were performed by the same physician who was proficient in arthroscopic techniques. After, the patient underwent tracheal intubation and general anesthesia. Those without contraindications use controlled blood pressure to control blood pressure at 100 mmHg/60 mmHg. A modified lateral position was taken, with the trunk tilted back 20–30°, shoulder abduction 30–70°, and flexion 15–20°. The surgical area was sterilized and draped, the sterile arm was pulled by 3–5 kg, the soft cushion under the armpit assisted in abduction, and the anatomical position and approach were marked. First, the conventional shoulder arthroscopy approach was adopted to explore the joint cavity, and the synovial hyperplasia in the joint cavity and the damage of various anatomical structures were observed. An anterior approach was established through the rotator cuff space under arthroscopic monitoring, and typical diseased synovial tissues were selected for pathological examination with the nucleus pulposus clamp. The synovium, articular cartilage denudation, and part of the diseased subchondral bone were removed, and the adhesion tissue in the shoulder joint was released. The shoulder joint has been moved in all directions, which can suck up the tissue debris in the joint cavity. When necessary, the anterior and posterior approach was used to enter the subacromial space for acromial formation and drainage of pus in the bursa. After the operation, the bleeding was completely stopped, the wound was sutured, and cotton pads were used to protect the underarms and then bandaged appropriately. Usually under arthroscopy, the synovial fluid is yellow and turbid, with varying degrees of synovial hyperplasia, hyperemia, edema, hypertrophy, paleness, granuloma and pannus, cartilage degeneration defect, cartilage exfoliation, subchondral bone exposure, and subchondral bone destruction. Nutritional support treatment is given after surgery, such as a high-protein diet. And it is necessary to take isoniazid, rifampicin, ethambutol, and pyrazinamide quadruple anti-tuberculosis treatment for 12 months. The shoulder joint abduction brace has been worn and fixed in the abduction and external rotation position for 6–8 weeks. After awakening from anesthesia, the patient was instructed to start wrist flexion and dorsiflexion activities and elbow joint flexion and extension activities. On the second day after the operation, the patient was instructed to perform mild shoulder joint passive abduction and external rotation function training, and the amount of activity was gradually increased 6 weeks after the operation until the maximum tolerance or nearly returned to normal. Blood routine, ESR, CRP, liver and kidney function, and other indicators after surgery were regularly reviewed and payed attention to prevent postoperative joint stiffness and muscle atrophy.

### Observation indicators and statistical analysis

Follow-up was 1 week, 4 weeks, and 3, 6, 12, 18, and 24 months after surgery. The VAS pain score, Constant score, ESR value, and CRP value were recorded before treatment and at the last follow-up. The statistical data were analyzed by SPSS 17.0 statistical software. The enumeration data was expressed with mean ± standard deviation. Before and after treatment VAS pain score, Constant score, ESR value, and CRP value were conducted by paired *t* test. The test level was 0.05. *P* < 0.05 indicated statistical significance.

## Result

All the patients had a smooth operation, the incision was healed in stage I, and no serious complications such as postoperative infection and nerve injury occurred. All 29 cases were followed up. The follow-up time was 12–24 months, with an average follow-up time of 15.7 months. The postoperative pathological diagnosis of 29 patients was shoulder tuberculosis. At the last follow-up evaluation, the VAS pain score, Constant score, ESR, and CRP were significantly improved compared to before treatment (Table 1).

**Table 1:** Comparison of results before treatment and at the last follow-up (*x* ± *s*)

	VAS score	Constant score	ESR (mm/h)	CRP (mg/L)
Before treatment	6.8 ± 1.3	50.0 ± 5.7	41.2 ± 7.6	19.7 ± 5.0
Last follow-up	1.2 ± 0.8	83.0 ± 4.8	11.5 ± 2.6	4.3 ± 1.4
Paired *t* test	*P* < 0.01	*P* < 0.01	*P* < 0.01	*P* < 0.01

## Case

**Fig. 1:**
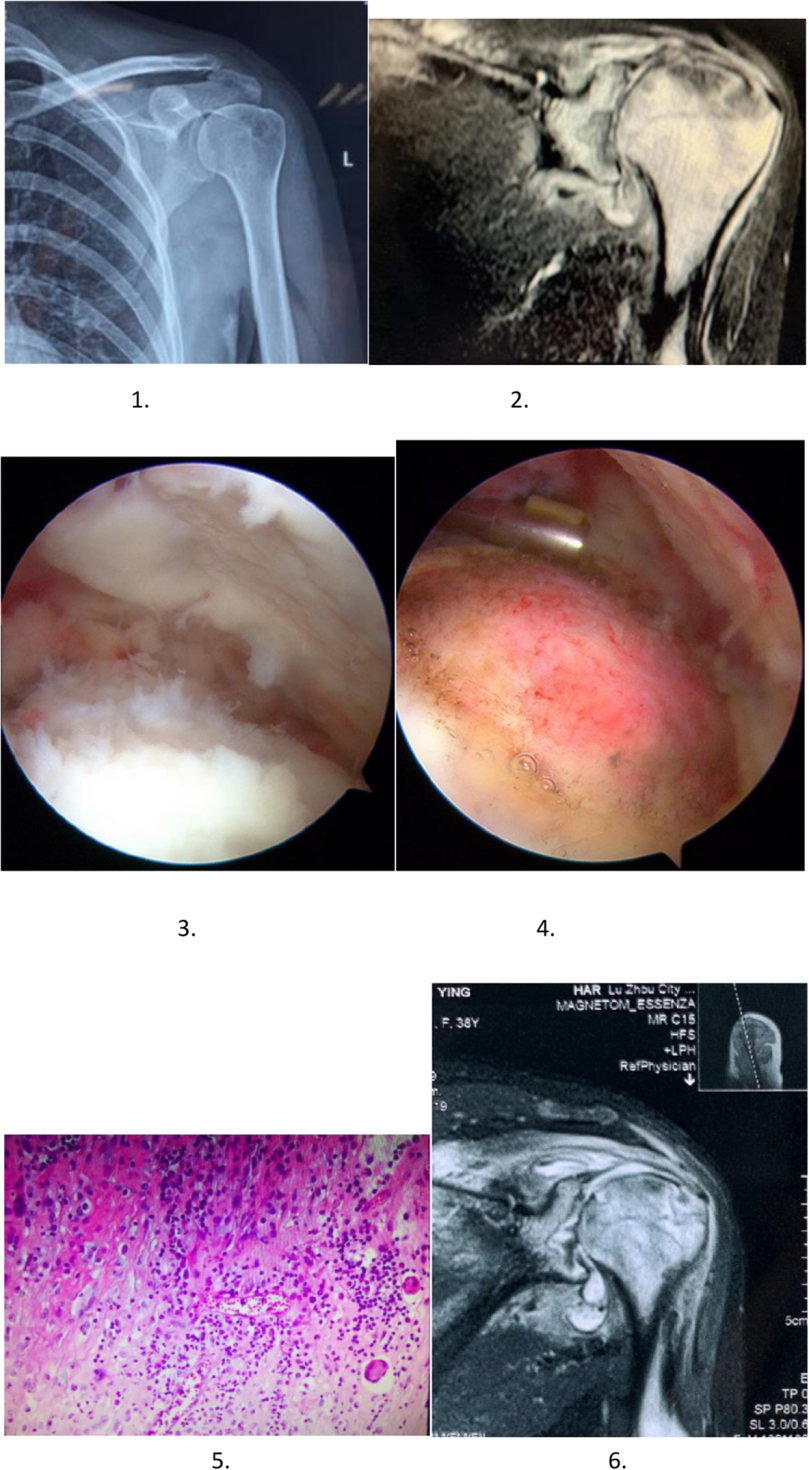
A 38-year-old female patient with joint tuberculosis of left shoulder. **1** Preoperative anteroposterior X-ray. **2** Preoperative coronal MRI. **3**, **4** Arthroscopic images. **5** Postoperative pathological sections. **l**, **6** Coronal MRI 2 months after surgery

A female, 36 years old, had left shoulder pain and limited mobility for 2 years. Physical examination revealed pain in the front and back of his left shoulder. The left shoulder joint is limited in abduction, adduction, internal rotation, and extension. The X-ray showed that the bone of the left shoulder joint was slightly damaged. MRI showed narrowing of the glenohumeral joint space, thickening of the glenohumeral joint synovium, effusion in the joint cavity, and swelling of the soft tissue around the joint muscles. The diagnosis of tuberculosis of the left shoulder joint has been considered. The symptoms were improved after 3 weeks of four anti-tuberculosis treatments. Shoulder arthroscopic debridement was performed 3 weeks later. During the operation, a large number of synovial hyperplasia, congestion, and swelling in the joint cavity were seen, and the articular cartilage of the humeral head was extensively destroyed, and the exfoliated cartilage was removed. Postoperative pathological examination confirmed shoulder tuberculosis. Regular anti-tuberculosis treatment was continued for 12 months after the operation. After 24 months of follow-up, the symptoms of the shoulder joint improved significantly (Fig. 1).

## Discussion

Tuberculosis caused by *Mycobacterium tuberculosis* has been accompanied by the development of human civilization. With the advent of anti-tuberculosis drugs, tuberculosis has gradually been effectively controlled. Bone and joint tuberculosis accounts for about 10% of extrapulmonary tuberculosis [3, 12]. Spine tuberculosis, hip tuberculosis, and knee tuberculosis account for the majority of bone and joint tuberculosis, while shoulder tuberculosis is rarely reported. In recent years, due to the increase in world population movement and the increase in drug-resistant strains, the incidence of tuberculosis is showing an upward trend. Especially in underdeveloped areas in western China, tuberculosis, especially bone and joint tuberculosis, is still relatively common [3]. This study uses arthroscopy to diagnose and treat patients and achieve significant results. It can not only completely clear the lesion, but also reduce the effect of inflammatory factors. It can also help undiagnosed patients before the operation to confirm the diagnosis through intraoperative observation and postoperative pathological examination. Shoulder arthroscopic surgery has minimal invasiveness, a wide field of vision under the arthroscope, thorough removal of lesions, and rapid functional recovery. Continuing to cooperate with anti-tuberculosis drug treatment can effectively control the development of the disease and protect joint function.

The clinical symptoms of shoulder tuberculosis are atypical. Most patients do not have the characteristic symptoms of tuberculosis such as hot flashes, night sweats, and cough. Moreover, most patients failed to find a history of tuberculosis. Shoulder tuberculosis often has no specific manifestations on imaging and laboratory indicators and often needs to be distinguished from shoulder joint purulent infection, shoulder joint malignant tumors, rheumatoid arthritis, gouty arthritis, and pigmented villonodular synovitis [13–18]. Diagnostic puncture and extraction of joint fluid applied to shoulder tuberculosis often results in negative results and needs to be performed multiple times. Not only does it not help diagnose, but also increases the risk of joint cavity infection and tuberculosis dissemination. Furthermore, clinical orthopedic doctors do not have a deep understanding of shoulder tuberculosis, and they often fail to consider the possibility of tuberculosis. The onset of shoulder tuberculosis is insidious, and many patients do not seek medical attention until they have joint dysfunction or joint swelling and pain. Therefore, early diagnosis and treatment are often not available, which causes a prolonged course of shoulder tuberculosis patients, and the disease continues to develop and spread. From the early stage, simple synovial tuberculosis or subchondral bone destruction develops into total joint tuberculosis. It even involves surrounding muscles, tendons, nerves, and lymph nodes. The disability rate is very high, which seriously affects the quality of life of patients [14].

The early diagnosis and early treatment of shoulder tuberculosis are for the recovery of the patient’s limb function and the improvement of symptoms. In daily diagnosis and treatment activities, clinicians should pay attention to inquiring about the patient’s medical history and grasp every information that is helpful for diagnosis: the patient’s past history, with or without tuberculosis poisoning symptoms or tuberculosis-specific signs, such as cough, night sweats, low fever, fatigue, weight loss, etc. In some cases, recurrent shoulder swelling and pain with limited mobility are present. In imaging examinations, MRI is the most important auxiliary diagnostic method. MRI of shoulder tuberculosis generally manifests as roughness and narrowing of the joint space, thickening of synovial edema, joint capsule effusion, discontinuous bone cortex, increased bone signal, bone quality irregular destruction, etc. When a highly suspicious case is found, diagnostic anti-tuberculosis treatment can be performed. The diagnosis of shoulder tuberculosis should be based on the patient’s clinical symptoms, medical history and signs, imaging findings, and laboratory indicators to make a comprehensive consideration. Pathological diagnosis is the gold standard of shoulder joint diagnosis. In this study, the minimally invasive method of shoulder arthroscopy can obtain pathological examination specimens as soon as possible, which has a high utility value.

In recent years, with the continuous development and maturity of shoulder arthroscopy technology, shoulder arthroscopy technology has been widely developed in orthopedics clinics. Compared with the open operation, arthroscopy has the advantages of being minimally invasive, having wider, clearer vision and larger cleaning area under the arthroscope, and thorough removal of the lesion. However, there have been few reports on the treatment of shoulder tuberculosis by arthroscopy. In other joint tuberculosis, the treatment with arthroscopy has achieved good results [19–21]. This is similar to the clinical results achieved in this study. Shoulder arthroscopy can effectively control the development of tuberculosis through a thorough and effective removal of necrotic tissues and drug treatment, so as to achieve good therapeutic effects.

The early stage of shoulder tuberculosis usually manifests as synovial tuberculosis and subchondral tuberculosis. Synovial tuberculosis spreads from the blood source and infects the synovium, causing synovial congestion and swelling into villonodula and synovial tissue hyperplasia. Sometimes granuloma or pannus can be seen. Due to the poor blood supply to the cartilage, *Mycobacterium tuberculosis* invades the joints and spreads along the subchondral area, causing subchondral bone destruction and cartilage denudation.

Early diagnosis or high suspicion of shoulder tuberculosis can be roughly diagnosed by observing synovial tissue and cartilage denudation under an arthroscope after a period of anti-tuberculosis treatment. After obtaining the synovial tissue and other specimens, perform a pathological examination to achieve the purpose of confirming the diagnosis. At the same time, the hyperplastic synovium, fibrous necrotic tissue, cartilage denudation, and tuberculosis foci are removed, and the adhesions are loosened under the arthroscope, and the dead space is eliminated. Continuous lavage of the joint cavity under the arthroscope can not only completely remove the necrotic tissue and inflammatory factors, but also improve the internal environment of the joint and effectively prevent the recurrence and spread of tuberculosis [22, 23]. For end-stage shoulder tuberculosis, since it has developed into total joint tuberculosis, a large amount of bone has been destroyed by tuberculosis, arthroscopy can still remove dead bones, synovial tissue, and tuberculosis foci; reduce inflammatory mediators; and slow the progression of the disease, creating opportunities for other operations and treatments for the future and increasing the success rate of operations and the cure rate of diseases. Arthroscopic treatment of shoulder tuberculosis is a relatively new diagnosis and treatment method, which is not well known to the majority of clinical orthopedic surgeons. To improve the diagnosis and treatment of shoulder tuberculosis requires the communication and cooperation among the departments of pathology, imaging, infection, and orthopedics [24, 25]. Enhancing clinician comprehension of all aspects of the disease and applying new treatment methods to clinical practice can ultimately benefit the patient [26]. Due to the small sample size in this study, it is impossible to compare drug therapy with arthroscopy diagnosis and treatment, and a larger number of patients will be needed for research in the future.

## Conclusion

Arthroscopic debridement is a minimally invasive treatment for shoulder tuberculosis, with complete debridement and short functional recovery time. Combined regular drug therapy can effectively treat shoulder tuberculosis with satisfactory results and is worthy of clinical promotion.

## Data Availability

The datasets analyzed during the current study are available from the corresponding author on reasonable request.

## Abbreviations

TB: Tuberculosis
MTB: *Mycobacterium tuberculosis*
CRP: C-reactive protein
ESR: Erythrocyte sedimentation rate
MRI: Magnetic resonance imaging
VAS: Visual analogue scale
WHO: World Health Organization
